# Deep Learning-Powered Prediction of Human-Virus Protein-Protein Interactions

**DOI:** 10.3389/fmicb.2022.842976

**Published:** 2022-04-15

**Authors:** Xiaodi Yang, Shiping Yang, Panyu Ren, Stefan Wuchty, Ziding Zhang

**Affiliations:** ^1^State Key Laboratory of Agrobiotechnology, College of Biological Sciences, China Agricultural University, Beijing, China; ^2^State Key Laboratory of Plant Physiology and Biochemistry, College of Biological Sciences, China Agricultural University, Beijing, China; ^3^Department of Computer Science, University of Miami, Miami, FL, United States; ^4^Department of Biology, University of Miami, Miami, FL, United States; ^5^Sylvester Comprehensive Cancer Center, University of Miami, Miami, FL, United States

**Keywords:** human-virus protein-protein interactions, machine learning, deep learning, transfer learning, prediction

## Abstract

Identifying human-virus protein-protein interactions (PPIs) is an essential step for understanding viral infection mechanisms and antiviral response of the human host. Recent advances in high-throughput experimental techniques enable the significant accumulation of human-virus PPI data, which have further fueled the development of machine learning-based human-virus PPI prediction methods. Emerging as a very promising method to predict human-virus PPIs, deep learning shows the powerful ability to integrate large-scale datasets, learn complex sequence-structure relationships of proteins and convert the learned patterns into final prediction models with high accuracy. Focusing on the recent progresses of deep learning-powered human-virus PPI predictions, we review technical details of these newly developed methods, including dataset preparation, deep learning architectures, feature engineering, and performance assessment. Moreover, we discuss the current challenges and potential solutions and provide future perspectives of human-virus PPI prediction in the coming post-AlphaFold2 era.

## Introduction

Currently, viral infection is a major factor threatening human health and global economic development (Qiu et al., 2017; Rasul, 2020; Lu and Peng, 2021). For instance, the current pandemic disease of novel coronavirus pneumonia, induced by the severe acute respiratory syndrome coronavirus 2 (SARS-CoV-2), has caused nearly 280 million confirmed cases and more than 5 million deaths worldwide by the end of 2021.^1^ Viruses invade host cells and complete their own life cycle by exploiting the host’s molecular machinery, which is largely determined by virus-host protein-protein interactions (PPIs) (Jean Beltran et al., 2017). Therefore, systematic characterization of human-virus protein interactions can help to decipher viral infection mechanisms and provide new leads for antiviral drug discovery and vaccine development. Experimental techniques [e.g., yeast two-hybrid (Y2H) assays (Calderwood et al., 2007; Tripathi et al., 2010; Rozenblatt-Rosen et al., 2012) and affinity purification coupled with mass spectrometry (AP-MS) (Shah et al., 2018; Gordon et al., 2020; Li et al., 2021; Stukalov et al., 2021)] have determined a great amount of human-virus protein interactions. Despite such tremendous progress in the last decades, human-virus interactomes are still far from complete, while existing interaction data usually focus on some well-studied virus species (Lian et al., 2021).

**FIGURE 1:**
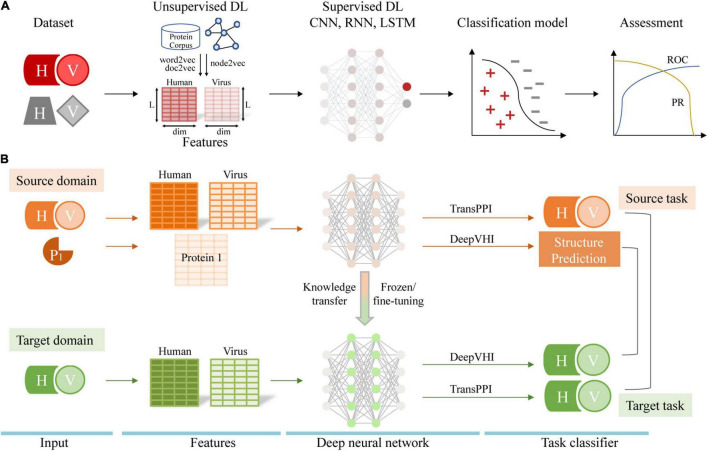
**(A)** Workflow of human-virus PPI prediction covering dataset construction, feature engineering, model construction, and performance assessment. ROC indicates receiver operating characteristic curve and PR indicates precision-recall curve. **(B)** Transfer learning for the human-virus PPI prediction task. H, V, and P1 represents human protein, viral protein, and the single protein, respectively.

To complement experimental methods, many computational methods have been developed to automatically predict PPIs between human host and various viruses. Existing prediction methods include interolog mapping (Yu et al., 2004; Yang et al., 2021a), domain-domain/motif interaction-based inference (Dyer et al., 2007; Evans et al., 2009; Chiang et al., 2017; Zhang et al., 2017), structure-informed method (de Chassey et al., 2013; Lasso et al., 2019) and machine learning (ML)-based prediction (Dyer et al., 2011; Barman et al., 2014; Yang et al., 2020). For more information on these computational methods, see the reviews (Mariano and Wuchty, 2017; Lian et al., 2021). With the accumulation of experimental PPIs, ML-based methods have been increasingly popular to predict human-virus PPIs. Briefly, ML-based methods train a binary classifier using known human-virus PPI data to predict interacting protein pairs from query samples. Traditional ML methods, such as support vector machines and random forests, have been used extensively and achieved reasonable performance (Emamjomeh et al., 2014). As an important branch of ML, deep learning (DL) has been successfully applied to predict intra-species protein interactions (Du et al., 2017; Hashemifar et al., 2018; Li et al., 2018; Chen et al., 2019). Very recently, several DL architectures have been developed to predict human-virus PPIs with favorable performance compared to traditional ML methods (Lanchantin et al., 2021; Liu-Wei et al., 2021; Tsukiyama et al., 2021; Yang et al., 2021b). In this review, we provide an overview of dataset construction, model architectures, feature engineering and performance assessment of DL in human-virus PPI identification (Figure 1A). In particular, we also discuss the technical challenges and future directions of this exciting topic in the coming era of post-AlphaFold2 (Jumper et al., 2021).

## Dataset Construction of Human-Virus Protein-Protein Interaction Prediction

### Positive Sample Selection and Filtering

The construction of training/test datasets, including positive and negative samples, is the first important step in developing a DL-based predictor. Generally, positive samples are experimentally determined human-virus PPIs, which can be collected from public database resources such as HPIDB (Ammari et al., 2016) and HVIDB (Yang et al., 2021a), or directly adopted from literature. Considering that experimental results may contain false positives, the obtained positive data should be further filtered according to various strategies. Both LSTM-PHV (Tsukiyama et al., 2021) and DeepViral (Liu-Wei et al., 2021) downloaded human-virus PPIs from HPIDB and only retained interactions with a significant MI score (a confidence score of molecular interactions) (Villaveces et al., 2015). In our previous works [i.e., TransPPI (Yang et al., 2021b) and doc2vec + RF (Yang et al., 2020)], we excluded interactions from large-scale MS experiments that have been experimentally detected only once to obtain a high-quality positive dataset. DeepVHPPI (Lanchantin et al., 2021) directly used the compiled dataset of our previous doc2vec + RF method (Yang et al., 2020). Still, the selection of high-confidence interactions is usually met with a tradeoff strategy between training data set size and quality as a perfect scoring system for assessing the reliability of experimental human-virus PPIs is still not available. While large known virus-host PPI data allow us to filter interactions with strict criteria, we can only adopt loose filtering criteria when only scarce interaction data are available to ensure that the retaining data size and quality are sufficient for training.

### Negative Sampling

In the absence of a gold standard for negative sample selection, random sampling is probably the most commonly used method (Dyer et al., 2011; Barman et al., 2014). For example, DeepViral randomly samples pairs of human and viral proteins that do not occur in the positive dataset (Liu-Wei et al., 2021). However, random sampling may inevitably introduce false-negative data points in the compiled training sets, prompting the development of a different negative sampling method called “Dissimilarity-Based Negative Sampling” (Eid et al., 2016; Yang et al., 2020, 2021a,b; Tsukiyama et al., 2021). The core idea is that if a viral protein *A* is similar to a viral protein *B* that interacts with human protein *C* (i.e., *B-C* is a positive sample), then the virus-host protein pair *A-C* cannot be a negative sample.

Another open issue related to negative sampling is the ratio of positive to negative samples. Often, a simple balanced ratio (i.e., 1:1) is used for many prediction tasks. However, it will cause the overestimation of model performance if the number of negative samples is obviously larger than that of positive samples in the real world (e.g., the issue of PPI prediction). An extremely unbalanced ratio will also yield biased results by over-predicting false negatives since negative samples are over-represented in the training set. Although a perfect solution for the ratio of positive-to-negative does not exist, an imbalanced ratio (e.g., 1:10) has been proven reasonable to predict human-virus PPIs (Yang et al., 2020; Liu-Wei et al., 2021; Tsukiyama et al., 2021).

## Deep Learning in Human-Virus Protein-Protein Interaction Prediction

### Classification Model Construction Through Supervised Deep Learning

In contrast to traditional ML methods, DL approaches are flexible in allowing the known labels to relate to the input feature vectors (Wainberg et al., 2018). However, the large number of trainable parameters in DL creates more challenges to avoid model overfitting (i.e., lose the generalization to new data) compared to traditional ML techniques. To deal with this issue, early stopping mechanisms by monitoring loss on the training and validation sets, regularization of the model, or dropout techniques are often adopted. As flexible architectures are a main feature of DL approaches, some dominant DL architectures such as convolutional neural network (CNN), recurrent neural network (RNN), long-short term memory (LSTM) have been used to predict human-virus protein interactions (Table 1). Such DL architectures can be considered feature extractors, which usually connect fully connected layers–also called Multi-layer perceptron (MLP)–to provide end-to-end binary classifiers for PPI prediction. After such supervised learning steps, trained models can be used to predict interactions from query human-virus protein pairs.

**TABLE 1:** Existing deep learning prediction methods of human-virus PPIs.

Method	Virus species	Input information	Embedding approach	Model architecture	Number of positive/negative samples	Negative sampling	URL
TransPPI (Yang et al., 2021b)	Multiple viruses	Protein sequences	PSSM	CNN + MLP + transfer learning	31,381/313,810	Dissimilarity-based negative sampling	https://github.com/XiaodiYangCAU/TransPPI/
DeepViral (Liu-Wei et al., 2021)	14 viral families	Protein sequences, functions, and disease phenotypes	one-hot and node2vec	CNN + MLP	24,678/246,780	Random sampling	https://github.com/bio-ontology-research-group/DeepViral/
LSTM-PHV (Tsukiyama et al., 2021)	All viruses	Protein sequences	word2vec	LSTM + MLP	22,383/223,830	Dissimilarity-based negative sampling	http://kurata35.bio.kyutech.ac.jp/LSTM-PHV/
DeepVHPPI (Lanchantin et al., 2021)	Multiple viruses	Protein sequences	one-hot	CNN + MLP + transfer learning	22,653/226,530	Dissimilarity-based negative sampling	https://github.com/QData/DeepVHPPI/
MTT (Dong et al., 2021)	Multiple viruses	Protein sequences	mLSTM	MLP + transfer learning	Multiple settings	Multiple settings	https://git.l3s.uni-hannover.de/dong/multitask-transfer/

#### Convolutional Neural Networks

Deep neural networks with one or more convolutional and pooling layers (i.e., CNNs) are usually applied to process image data to capture local pixelated features (Krizhevsky et al., 2012). In recent years, CNNs have been widely used to capture protein features in bioinformatics studies (Hashemifar et al., 2018), allowing the effective detection of local motif features of proteins that mediate protein interactions while following pooling layers reduce the dimensions of feature maps. Our previous work applied a sequence-based siamese one-dimensional (1D) CNN architecture to train a human-virus PPI classifier and achieved better performance than traditional ML methods especially in relatively large datasets (Yang et al., 2021b). In particular, we employed the siamese network (Bromley et al., 1993) to learn complex interaction relationships between human and viral proteins. The core idea of the siamese network is parameter sharing between two identical subnetworks (i.e., the human and virus protein input subnetworks) that can effectively capture the mutual influence of protein pairs (Chen et al., 2019). Liu-Wei et al. (2021) employed 16 1D-convolutional layers with a pooling layer and several dense layers to predict human-virus PPIs. Moreover, Lanchantin et al. (2021) applied a convolutional layer with multiple convolutional filters for neural network training. Different architectures of CNNs in these publications further demonstrate the flexibility of DL.

#### Recurrent Neural Networks and Long Short-Term Memory

The main application of RNNs is in natural language processing, such as machine translation (Sutskever et al., 2014) and speech recognition (Graves et al., 2013). In particular, recurrent layers allow the handling and integration of complex long-range sequential information. Like convolutional layers, recurrent layers also scan the input sequential data element by element but preserve previous output value (i.e., a memory of the earlier state) that are combined with the current input value to output a value of the current state. RNNs are useful to convert variable-length data to fixed-size representations as the inputs to the next fully connected layers for prediction tasks (Greener et al., 2022). In particular, the more advanced bidirectional gated recurrent unit (GRU) variant of RNNs has been used to predict intraspecies protein interactions, showing excellent performance in combination with a CNN (RCNN) (Chen et al., 2019). Yet, this deep learning framework did not allow more favorable predictions of human-virus PPIs compared to simple CNNs (Liu-Wei et al., 2021). Gradient explosion and disappearance will occur when RNNs propagate backward since there are long-term dependencies over the sequential series (Sun et al., 2020). As an advanced architecture of RNN, LSTM introduces the concept of cells and gates (an input gate, an output gate and a key forget gate) (Gers et al., 2000). LSTM cells can store long-term information while these gates regulate the information into cells. Recently, Tsukiyama et al. (2021) employed two LSTM subnetworks to transform the human and viral proteins-embedding matrixes into two fixed-length vectors as the input to subsequent fully connected layers to predict human-virus PPIs. The LSTM architecture mitigates the gradient explosion and disappearance problems of RNNs, effectively preserving long-term memory information of protein sequences.

### Feature Engineering in Deep Learning

Protein feature vectors used in DL models are often inferred from protein sequences, including simple residue position information, physicochemical properties, and evolutionary information of residues, such as one-hot encoding and position-specific scoring matrix (PSSM) (Table 1). Briefly, the one-hot method encodes each amino acid as a vector of length *n* that corresponds to the set of amino acid categories, allowing us to represent a protein sequence of length *L* as a *L* × *n* matrix with 0 and 1 entries. As a more fine-grained method to present protein features PSSMs capture evolutionary relationships between proteins. In particular, each amino acid (*n*) in the protein sequence of length *L* has a specific score, allowing an alternative representation of a protein sequence as a *L* × *n* matrix. Furthermore, some word embedding techniques from natural language processing have been adapted to represent proteins, which can automatically convert *k*-mer amino acids or proteins to fixed-dimensional feature vectors. Here, we mainly focus on these embedding techniques and their applications in the DL-based prediction of human-virus PPIs.

#### Word2vec and Doc2vec

Word2vec is a word embedding technique derived from natural language processing to obtain distributed representations of words through model training. Word2vec uses two-layer shallow neural networks to obtain feature vectors of words by using linguistic contexts, where two architecture choices including continuous bag-of-words (CBOW) and skip-gram (Le and Mikolov, 2014; Kimothi et al., 2016) are often used. Briefly, the CBOW model predicts the current word by using surrounding context words while skip-gram uses the current word to predict the surrounding words. In particular, a textual corpus is generally used to train the word2vec model to assign fixed-dimensional vectors to words, enabling that the words sharing common contexts and semantics in the training corpus are embedded close to each other (Kimothi et al., 2016). Such an embedding approach has been applied to represent protein sequences in several bioinformatics tasks. For instance, the unsupervised word2vec model trained from a corpus containing non-redundant proteins in the Swiss-Prot database and the resulting feature vectors of human and viral proteins were further used to train human-virus PPI prediction models (Tsukiyama et al., 2021). In this work, *k*-mers (i.e., *k* consecutive residues) in each sequence were regarded as single words, representing each protein sequence through multiple *k*-mers. The authors employed the CBOW architecture to train the word2vec model and optimally set *k* to 4. As a result, 128-dimensional embedding vectors for multiple *k*-mers were retrieved and further concatenated to obtain embedding feature matrixes of proteins. Additionally, domains or motifs in proteins can also be treated as words in documents. Similar to the word2vec model, protein sequences can therefore be represented by feature vectors based on their domains or motifs (i.e., domain or motif embeddings). In Pan et al. (2021), the authors employed the skip-gram model to pre-train domain embeddings and averaged multiple domain embeddings in a protein sequence to construct the corresponding protein feature vector. The resulting protein feature vectors were further used to predict protein toxicity. Considering that human-virus protein interactions are generally mediated by domain-domain/motif interactions, the feature representation strategy of domain/motif embeddings should be informative in predicting human-virus PPIs.

As an extension of word2vec, doc2vec adds the whole document as another word. Doc2vec considers the context information of words and the whole document. When applied to protein sequences, each sequence is regarded as a document, in which *k*-mers are defined as the corresponding words (Yang et al., 2018). Subsequently, the doc2vec model is trained to learn the feature vector representation of each protein sequence in the corpus by using similar model architectures in word2vec. In our previous work (Yang et al., 2020), we successfully employed the doc2vec model to pre-train the embeddings of proteins based on the Swiss-Prot corpus. We further used the obtained low-dimensional feature vectors of human and viral proteins as input to train an RF classifier to predict human-virus PPIs (i.e., doc2vec + RF) and achieved better performance than other sequence-based traditional ML algorithms.

#### Node2vec

Graphs, also known as networks, have been widely used to represent biological entries (i.e., nodes) and their relations (i.e., edges). A series of graph embedding methods have been developed to automatically learn low-dimensional feature representation for each node in the graph (Grover and Leskovec, 2016; Ou et al., 2016; Wang et al., 2016). Such low-dimensional feature representations preserve the network structure information of the graph, which can be employed to train ML models to tackle node classification or link prediction problems (Yue et al., 2020). As one of the most commonly used graph embedding methods, node2vec firstly adopts a flexible random walk process to generate node sequences (multiple word lists), which are subsequently fed to the word2vec model to obtain node embedding features (i.e., node representations) (Grover and Leskovec, 2016). In the field of bioinformatics, node2vec is often used in node classification tasks such as identifying essential proteins based on a PPI network (Zeng et al., 2019) and detecting tissue-specific cellular functions through multi-layer PPI networks (Zitnik and Leskovec, 2017). Additionally, node2vec has been employed to obtain protein features based on the network consisting of proteins, Gene Ontology (GO) terms, and their associations called GO2Vec (Zhong et al., 2019). Further, these network embeddings were used to predict protein interactions (Zhong and Rajapakse, 2020; Liu-Wei et al., 2021). In particular, Liu-Wei et al. (2021) employed their DL2Vec method (node2vec variant) to embed human and viral proteins by using GO and cross-species phenotype ontology annotations. Such embeddings were then used as input to train a neural network, allowing the reliable prediction of human-virus PPIs, suggesting that node embedding is informative in recognizing human-virus PPIs.

## Model Generalization Through Transfer Learning

Since data available for training human-virus PPI prediction models of novel or rarely investigated virus species are often limited, the lack of sufficient labeled data is a major obstacle to ML-based PPI identification. Transfer learning is a good solution for processing relatively scarce data and improving prediction performance. The core idea of transfer learning is to leverage informative prior knowledge learning from other related tasks to enable learning of a target task with small-scale data. In the context of DL, deep transfer learning is becoming a promising method in generalizing a DL-based human-virus PPI prediction model.

Our recent work, TransPPI, employed two transfer learning approaches to accurately predict human-virus PPIs (Yang et al., 2021b). Specifically, we trained a CNN (i.e., the feature extractor) as well as fully connected layers (i.e., the MLP classifier) with multiple large-scale human-virus PPI datasets. In the next step, we retrained the model on the target human-virus PPI dataset through two types of transfer learning. (i) In the “frozen” approach, we kept learned parameters of CNN layers unchanged and retrained MLP layers with a target dataset; (ii) In the “fine-tuning” approach we retrained both CNN parameters and MLP layers with a target dataset. In general, the above transfer learning strategies effectively utilized prior knowledge from a “source” (e.g., human-HIV PPIs) to train in a target task domain (e.g., human-SARS-CoV-2 PPIs), allowing us to improve the performance and generalization of models based on small-scale data (Figure 1B).

In a different approach, Lanchantin et al. (2021) adopted a new transfer learning strategy to predict human-virus PPIs for a novel virus without any experimental known interaction data. The proposed architecture called DeepVHPPI first pre-trained supervised structure prediction (i.e., secondary structure prediction, residue contact prediction and remote homology detection) models as source tasks. Then, their approach fine-tunes the entire neural network on the target task (human-virus PPI prediction) by transferring information from source tasks (Figure 1B). Finally, DeepVHPPI showed promising prediction performance when determining interactions with human-SARS-CoV-2. While the underlying principle is based on the assumption that both source and target learning objectives share similar statistical properties, allowing to share similar model parameters, the transfer learning strategies of TransPPI and DeepVHPPI are different. In particular, TransPPI transfers model parameters that were learned from a source, large-scale human-virus PPI data set to predict interactions in a different target human-virus setting. In comparison, DeepVHPPI transfers feature representations of protein structures that were learned from a source data set to predict human-virus PPIs, assuming that the sequence-structure relationship of interacting proteins is similar, regardless of the considered organisms.

Dong et al. (2021) employed a multi-task transfer learning method called MultiTask Transfer (MTT) to construct human-virus PPI prediction model for novel viruses. Using a pre-trained UNIREP model (Alley et al., 2019) based on multiplicative LSTM (mLSTM) human and viral protein embeddings were obtained to predict human-specific and human-virus PPIs based on known PPI data from various benchmark datasets. In particular, such an approach makes the implicit assumption that the underlying statistical characteristics of amino acid composition of interacting proteins are generally similar. Although viral proteins try to mimick human interaction partners to bind to a specific host protein (Mariano and Wuchty, 2017), the number of human interaction partners a virus usually interacts with is rather limited. As human interaction partners hardly cover the whole human proteome, human PPIs potentially introduce a training bias, overpowering the specificity of human-virus interactions.

## Strength and Weakness of Existing Deep Learning-Based Human-Virus Protein-Protein Interaction Prediction Methods

In Table 1, we summarize recently developed deep learning-based human-virus PPI prediction methods (see Sections “Dataset Construction of Human-Virus Protein-Protein Interaction Prediction” to “Model Generalization Through Transfer Learning” for details of methods) to further analyze the strengths and weaknesses of these methods. LSTM-PHV employed word2vec + LSTM + MLP framework to train the human-virus PPI prediction model, where word2vec effectively captures context semantic information of *k*-mer amino acids. Furthermore, LSTM mitigates the explosion and disappearance of gradients in RNNs, enabling long-range sequential learning. Notably, other methods mainly adopt CNNs in their model architecture, better capturing local features of protein sequences, such as linear binding motifs that mediate human-virus PPIs compared to RNN/LSTM-based methods.

As the main innovation, DeepViral learns protein representations, that account for GO and disease phenotype ontology information as additional features to simple sequence information using a node2vec approach. Although such an approach allows a better representation of proteins compared to a simple one-hot sequence representation, this feature encoding method comparatively relies on functional and disease phenotype data of human and viral proteins. Such a dependence on auxiliary data may be limiting the method applicability to host-virus domains where virus specific information is missing.

The highlight of TransPPI and DeepVHPPI is the application of transfer learning techniques, that can improve model performance and generalization ability when available training data of novel or rarely investigated virus species are limited. In contrast to DeepVHPPI, TransPPI taps similarities of sequence composition of interacting human and viral proteins, potentially leading to better prediction performance. In particular, DeepVHPPI trains on a human-all virus PPI set, which is finally used to predict human-specific virus PPIs. In contrast, TransPPI requires that the target virus species has a small number of known human-virus PPI data. DeepVHPPI does not have this requirement, making this approach applicable to host-virus pairs where no experimental data is available. Another transfer learning method MTT mainly employs a multi-task learning strategy by considering human-specific PPIs as well. While such auxiliary training data improves model generalizability, such PPIs also introduce host-specific interaction characteristics that may impair the specificity to detect host-virus interactions.

## Discussion

Deep learning is playing an increasingly important role in human-virus PPI prediction. Although existing DL methods have outperformed traditional ML methods in predicting human-virus PPIs, much room for improvement remains. First, more DL architectures and feature representations should be used. The optimal combination of the DL architecture and feature engineering should be sought to maximize prediction performance. Existing DL methods may supplement previous human-virus PPI prediction methods. Thus, the integration of different prediction methods can often result in a more accurate and robust predictor. Moreover, model interpretability received a wide concern for ML-based methods. Usually, the way DL architectures end up with their predictions and predictive features are unknown, prompting the call for more explainable DL methods. In some bioinformatics tasks (Pan et al., 2021; Zhu et al., 2021), the prediction models have generally been simply interpreted by using t-distributed stochastic neighbor embedding (t-SNE) (van der Maaten and Hinton, 2008) to visualize the learned high-dimensional feature representations in 2D space. Note, that such t-SNE-based visualization can merely demonstrate the general effectiveness of the feature embedding methods, while the contributive features are not highlighted. Recently, attention mechanisms have provided a new direction for interpreting black-box DL models (Choi et al., 2017; Zhou et al., 2018), which should be introduced to interpret the DL models of human-virus PPI predictions as well.

Similar to other bioinformatics prediction tasks, rigorous and fair performance comparison of different human-virus PPI prediction methods is crucial. Generally, the performance of a newly developed human-virus PPI prediction is evaluated by using test sets that are specifically compiled or commonly used (Barman et al., 2014; Eid et al., 2016). Considering that such datasets were constructed based on different criteria, the performance comparison of different methods will inevitably yield biased results. To allow a more comprehensive method comparison, community-wide efforts should be taken. First, some comprehensive human-virus PPI data sets with strict reliability and quality controls should be compiled, which is fundamental for comparing different methods. Second, the developers should make their methods freely accessible to the community either through the construction of web servers or the release of source codes. Third, third-party teams should be encouraged to conduct a critical assessment of different prediction methods to obtain more unbiased comparison results. Last but not least, regular community-wide competition is also helpful to boost the improvement of human-virus PPI prediction. To this end, we should follow the successful experience of the Critical Assessment of protein Structure Prediction (CASP) experiments.^2^

Currently, dramatic progress in protein structure prediction has been made by AlphaFold2, a DL-powered method developed by the research team of DeepMind, and its high-accuracy performance has been reported in the CASP14 experiment (Jumper et al., 2021). Undoubtedly, the coming post-AlphaFold2 era will provide an unprecedented opportunity for the protein bioinformatics community, suggesting that many prediction methods can be significantly improved and upgraded by incorporating accessible and accurate structural information, including the prediction of human-virus PPIs. First, structural information has been widely used in previous human-virus PPI prediction methods. For instance, the P-HIPSTer model developed by Lasso et al. (2019) relied on the structural similarity of query human-virus protein pairs to known structural domain-domain/motif interactions to quantify the interaction possibility of query protein pairs. Although P-HIPSTer provided accurate prediction results, coverage of the predicted interactome is insufficient, mainly as a consequence of limited available 3D structures. With more accurate structural predictions from AlphaFold2, prediction coverage of such structure-informed human-virus PPI prediction method can be significantly increased. Second, the available structural information can contribute rich feature representations to develop DL-based prediction models. For instance, residue-level structural features can be easily introduced into the established DL architectures. 3D structures of proteins can also be converted into graphs, allowing the application of more effective DL architectures such as graph convolutional neural networks. Last but not least, highly accurate protein structures will not only propel binary PPI predictions but also predict interaction details from binding regions/residues to 3D conformational dynamics of two interacting proteins. Indeed, Baek et al. (2021) have taken the initiative to employ two DL-based structure prediction methods (i.e., RoseTTAFold and AlphaFold2) to systematically detect PPIs and construct accurate 3D models of protein complexes within the yeast proteome (Humphreys et al., 2021), which will be used for human-virus PPI prediction as well in the future. Very recently, Gao et al. (2022) developed a DL-based protein complex prediction method termed as AF2Complex, in which AlphaFold2 monomer models were employed to predict the structures of multimeric protein complexes and metrics for predicting direct PPIs between arbitrary protein pairs were also introduced. Considering AF2Complex does not rely on paired multiple sequence alignments, it could be suitable for addressing human-virus PPIs. Taken together, we are fast approaching the development of successful methods to predict human-virus PPIs empowered by DL and AlphaFold2, unveiling the secrets of human-virus relationships.

## Data Availability Statement

The original contributions presented in the study are included in the article/supplementary material, further inquiries can be directed to the corresponding author.

## Author Contributions

XY wrote the draft of the manuscript. ZZ supervised the work and significantly revised the manuscript. SW, SY, and PR revised the final version of manuscript. All authors contributed to the article and approved the submitted version.

## Conflict of Interest

The authors declare that the research was conducted in the absence of any commercial or financial relationships that could be construed as a potential conflict of interest.

## Publisher’s Note

All claims expressed in this article are solely those of the authors and do not necessarily represent those of their affiliated organizations, or those of the publisher, the editors and the reviewers. Any product that may be evaluated in this article, or claim that may be made by its manufacturer, is not guaranteed or endorsed by the publisher.

## Footnotes

1
https://covid19.who.int/

2
https://www.predictioncenter.org/
